# Has the prevalence of stunting in South African children changed in 40 years? A systematic review

**DOI:** 10.1186/s12889-015-1844-9

**Published:** 2015-06-05

**Authors:** Rihlat Said-Mohamed, Lisa K Micklesfield, John M Pettifor, Shane A Norris

**Affiliations:** MRC/Wits Developmental Pathways for Health Research Unit, Department of Paediatrics, Faculty of Health Sciences, University of the Witwatersrand, 7 York Rd, Parktown 2193, Johannesburg, South Africa

**Keywords:** Stunting, Undernutrition, Prevalence trends, Preschool children, South Africa, Sub-Saharan Africa

## Abstract

**Background:**

In the last 20 years, South Africa has experienced political, economic, and demographic transitions accompanied by an epidemiological transition. Like several sub-Saharan countries, the South African population is facing both under–and over–nutrition, and nutrition and lifestyle related chronic disease while the burden of infectious disease remains high. It is critical to understand these trends overtime in order to highlights the pitfalls and successful measures initiatives taken in the efforts to tackle malnutrition. The objective of this systematic review is to investigate the changes in the prevalence of stunting, a chronic form of undernutrition, in South Africa over 40 years, and to derive lessons from the South African experience, a country in an advanced process of transition in sub-Saharan Africa.

**Methods:**

We undertook a systematic review of publications selected from PubMed, Science Direct and Scopus. We included studies and surveys published between 1970 and 2013 if they reported the prevalence of stunting (low height-for-age) in children under-6 years of age living in South Africa. We excluded studies conducted in health facility outpatients or hospital wards, or children with known chronic and acute infectious diseases. We extracted Date of data collection, study setting, ethnicity, age, sex, sample size, growth references/standards, diagnostic criteria for stunting and prevalence of stunting from each study.

**Results:**

Over the last decade, the national prevalence of stunting has decreased. However, between and within provincial, age and ethnic group disparities remain. Unlike other countries in sub-Saharan Africa, no sex or rural/urban differences were found in preschool children. However, the analysis of long-term trends and identification of vulnerable groups is complicated by the use of different growth references/standards and sampling methods.

**Conclusion:**

Despite economic growth, political and social transitions, and national nutritional programs, stunting remains stubbornly persistent and prevalent in South Africa. A multi-sectoral and public health approach is needed to: (i) better monitor stunting over time, (ii) combat malnutrition during the first thousand days of life through continued efforts to improve maternal nutrition during pregnancy and infant feeding practices.

**Electronic supplementary material:**

The online version of this article (doi:10.1186/s12889-015-1844-9) contains supplementary material, which is available to authorized users.

## Background

In 2000, the United Nations Millennium Declaration committed 189 countries to halve the prevalence of underweight (weight-for-age < −2SD of the median of the reference population) in children under-five years old [1]. Although underweight has been reduced by 26 % between 1990 and 2011 in Sub-Saharan Africa (SSA), the progress towards meeting the Millennium Development Goal 1 (MDG1) target has been limited since differences remain between rural and urban areas (43 % vs. 30 %), low–and high-income households (48 % vs. 25 %), and females and males (36 % vs 42 %) [2].

Underweight does not distinguish between two different forms of malnutrition: (1) linear growth delay (stunting) due to chronic poor health and undernutrition; and (2) wasting associated with acute nutrient deprivation [3–6]. In 2008, the UN Standing Committee on Nutrition recommended that: “*progress towards the achievement of MDG1 should be reported against reductions in the prevalence of stunting* […]*, not just underweight*” [7]. In SSA, in 2011, 21 % of children under-five years old were underweight *versus* 40 % stunted [2, 8]. Stunting is defined as having a height-for-age index more than two standard deviations below the World Health Organization Child Growth Standard median [2, 9]. Determinants of stunting are multi-factorial and mainly associated with households’ low socio-economic status and food insecurity, repeated infectious episodes in infants and maternal health before, during and after pregnancy [2, 10]. Stunting presents a significant public health concern due to increased morbidity and mortality during the life course. Being stunted has been associated with delayed cognitive development, impaired physical growth, lower productivity, and a greater risk of poor health including the development of cardio-metabolic disease that may be transmitted to the next generation [11]. These short and long-term adverse effects on health and economics at individual, household and community levels highlight the need for reducing the prevalence of stunting [10]. In 2012 the WHO set Global Targets for Maternal, Infant and young Child Nutrition [12], the first of which is to reduce the number of stunted children under-5 years of age by 2025 by 40 %.

In comparison to other countries of SSA, South Africa differs by: its long history of recording nutrition data through studies and national surveys, and its advanced stage of nutrition transition associated with political, economic and demographic transitions [13]. These last 20 years, South Africa has experienced rapid nutrition and lifestyle transitions associated with increased prevalence of obesity and non communicable disease as experienced by western countries [13]. However, the South African pattern of transitions differs in that stunting persists and, in addition to other factors, may fuel the epidemiologic trends of the degenerative disease aforementioned. As a result, understanding the long term dynamic of stunting in South Africa is required in order to efficiently orientate public health interventions and reduce the consequences of stunting on population health and economic development.

The main objective of this systematic review is to understand the trends in the prevalence of stunting between 1970, when the first articles [14, 15] reporting the prevalence of low height in children were published; and 2013, when the results of the last national nutritional survey [16] were published which is 2 years before the date for achieving the Millennium Development Goals [1]. In particular, this systematic review aims to: (1) provide an overview of the changes in the prevalence of stunting in South Africa in children under-six years old since 1970; (2) identify the vulnerable populations and locations; and (3) derive lessons from the South African experience.

## Methods

The PRISMA Statement [17], evidence-based guidelines that provide a set of items to report in systematic reviews and meta-analyses, was used (Additional file 1).

### Eligibility

Studies published between 1970 and August 2013, irrespective of language, that report the prevalence of stunting or a low height-for-age in children under-six years old in South Africa, irrespective of the growth curves and the cut-off points used, were included. Studies conducted in clinical outpatients or wards of health facilities, or on children with chronic or acute diseases (e.g., HIV, tuberculosis) were excluded due to representativeness limitations.

### Identification of studies

Combinations of the following search terms were used in PubMed, Science Direct and Scopus: Stunting/-ed, Growth retardation, Height, Undernutrition, Malnutrition, South Africa, Humans, Under-five, Preschool, and Infant.

### Study selection

Firstly, we used titles and abstracts to select the studies. When the abstracts were not available, we kept the studies for the next level of screening. Secondly, articles were read entirely. We included the studies that matched our inclusion criteria. When we excluded a study, reasons were recorded. When we identified studies that might have been appropriate while reading, the studies were searched for. When several studies used the same data, we included the most recent publication. For longitudinal studies that reported the prevalence of stunting in their cohort at different ages in different articles, we included the publication that reported the prevalence at 2–3 years of age, as in many low and middle-income countries growth faltering occurs during the first 2 years of life [18].

### Data extraction

The following information were recorded in a database: date of data collection, study settings and designs, ethnicity, age, sex, sample size, reference/standard used to determine cut-off points, actual cut-off points for stunting, and the prevalence of stunting. Supplementary information were also recorded (e.g., objective of studies).

### Quality assessment

To our knowledge, tools for the quality assessment of prevalence studies on malnutrition do not exist [19]. Therefore, the tool developed for studies on the prevalence of temporomandibular disorders [20] was used here because it allows for the calculation of a *Total Quality Score* (TQS). This was obtained for each study by summing the points assigned for each of the items included in the following sections: sampling; measurement; analysis. The TQS range was from 0 (very bad) to 20 (outstanding) points.

As essential criteria in the assessment of stunting in children are the accuracy of the measurement of height and age, the items in the measurement section had to be adapted as follows:

- Reliability was assessed by the adequacy of the method used to measure height, and scored: 0, if the equipment used was not described; 1, if it was described and 2, if it was described, calibrated, and an inter-reliability coefficient was provided;
- Validity was assessed by the accuracy of the respondents’ age recorded and scored: 0, if the source of the child’s age was not provided; 1, if the age was provided by schools/parents, and 2, if the age was recorded from Road-to-Health card/clinical records/birth certificate.

### Data analyses

Publications were heterogeneous in terms of:

i. *Growth references/standards.* From 1970 to 2013, a variety of growth references/standards were used, namely Harvard [21], NCHS [22], CDC [23] and the WHO [9]. Since they were developed based on different population and selection criteria; it is inappropriate to compare the prevalence of stunting between studies using different growth references/standards. Thus the differences in prevalence of stunting between various studies were only compared if the same growth reference was used.
ii. *Definition of stunting.* Stunting was defined variably (height-for-age <80 % or <90 % of the median, or <3^rd^ or <5^th^ percentiles, or–2SD from median of the reference population). Since the reference values for height-for-age follow a Gaussian curve, cut-off points defined as percentiles and z-scores are very close to each other [24, 25] allowing comparisons.
iii. *Representativeness*. Data were separated according to the region covered, namely national, provincial, urban or rural area.
iv. *Ethnic groups.* Ethnic groups are used as reported in the articles and are consistent with the South African census terms [26]: black (African), coloured (mixed ancestry), white (European) and Indians (Asians from the subcontinent).
v. *Age and sex.* Where studies reported the prevalence for different age ranges or sex, the prevalence for the whole sample (both sexes or all ages) was calculated provided the sample size for each subgroup was given.
vi. *Year.* Prevalence were dated according to the year of data collection, when provided, otherwise the year of publication was used.

A Z-test was used to compare the prevalence of stunting between time points where appropriate (e.g., between surveys using the same reference/standard).

## Results

**Fig. 1:**
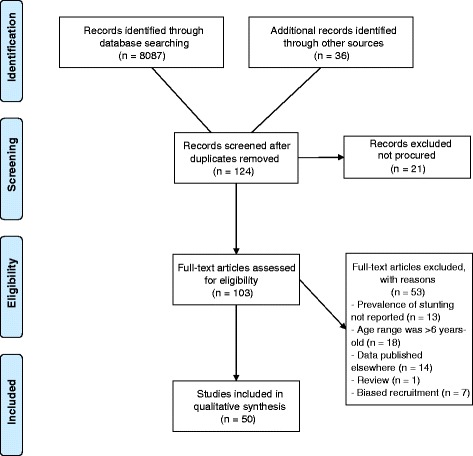
Flow Diagram

Eight thousand one hundred twenty three references were identified using the defined electronic databases (Fig. 1). Among them, 124 references whose titles or abstracts mentioned our search terms or reported the prevalence (or percentage) of at least one of the forms of malnutrition (*undernutrition*, *wasting*, *underweight*) were selected and read entirely. Of the 124 references, 21 could not be procured (Additional file 2) despite searches (internet, University of the Witwatersrand libraries, contact with The South African Nutrition Society). Publications were excluded because: the prevalence of stunting was not reported (*n* = 13), the age range was >6 years of age (*n* = 18), data published elsewhere were used (*n* = 14), the reference was a review (*n* = 1), or there was potentially biased recruitment (*n* = 7). Fifty studies were included in the systematic review (Additional file 3).

### Description of publications (Additional file 2)

Nine national surveys [16, 27–34], 39 cross-sectional [14, 15, 35–71] and one longitudinal [72, 73] studies were reviewed. Eight used the Harvard references, 31 used NCHS references, and four used the WHO standards. One study compared NCHS, CDC and WHO references, and one study compared NHANES I and II references. For five articles, the growth reference/standard used could not be discerned [43, 57–60].

We found three studies of poor quality (TQS ≤ 4), 13 of moderate quality (5 ≤ TQS ≤ 9), 12 of good quality (10 ≤ TQS ≤ 14) and 16 of outstanding quality (TQS ≥ 15). The aims of studies were: assessment of children’s nutritional status [14, 15, 36, 40, 41, 43, 45, 50–54, 57, 60, 66, 68–73], study of children’s growth patterns [37, 38, 43, 63, 71, 73], baseline of intervention studies or programs [14, 39, 42, 44, 46, 48, 50, 53, 55, 56, 59–62, 65, 67, 69], assessment of the relationship between ecological factors and children’s health status [15, 35, 36, 41, 47–49, 62, 67, 68, 70, 72].

### Evolution of the number of publications

**Fig. 2:**
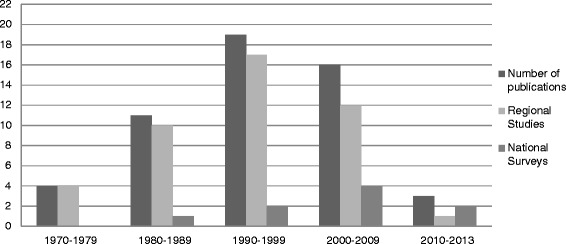
Total number of publications, number of regional studies and number of national surveys included in the systematic review, per decade

The first publications documented dates from the mid-1970’s [14, 15] (Fig. 2). The period from 1980 to 1999 is characterized by a progressive increase in the number of regional studies while the number of national surveys slowly increased from 1990 to 2010.

### Trends

**Fig. 3:**
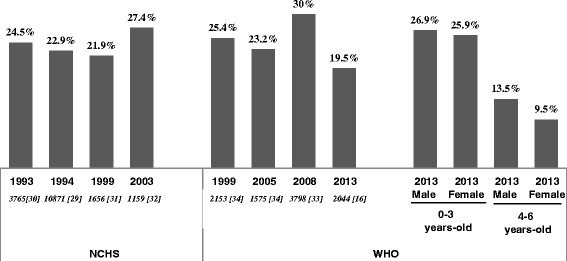
National prevalence of stunting in South Africa 1993–2013. Living Standards and Development Survey (1993) [30]; SA Vitamin A Consultative Group (1994) [29]; National Food Consumption Survey (1999) [31, 34]; Demographic and Health Survey (2003) [32]; National Income Dynamic Survey (2008) [33]; National Food Consumption Survey (2005) [34]; South Africa first National Health And Nutrition Examination Survey (2013) [16]. Sample size *in italics [reference number]*, sample size of male and female 0–3 years-old = 537 and 553, respectively; sample size of male and female 4–6 years-old = 503 and 451, respectively

The national prevalence of stunting is illustrated in Fig. 3. Using the NCHS reference, from 1993 [30] to 2003 [32] the prevalence of stunting increased by 2.9 % (z-test, p < 0.05). Using the WHO standard, the prevalence of stunting decreased by 5.9 % between 1999 [34] and 2013 [16] (z-test, p < 0.001). However, the 2008 National Income Dynamic Study (NIDS) showed an increase of 6.8 % from the 2005 National Food Consumption Survey (z-test, p < 0.001). A significant difference in the prevalence of stunting was found on data from the 1999 National Food Consumption Survey using the NCHS [31] and WHO [34] references/standards (21.9 % *vs*. 25.4 %; z-test, p < 0.05). This comparison illustrates the concerns around the use of different growth references [73]. In 2013 [16], the prevalence of stunting was higher in 0–3 years old than in their 4–6 year old peers (z-test, p < 0.001 within both sexes). The prevalence was similar between sexes at each time point.

**Fig. 4:**
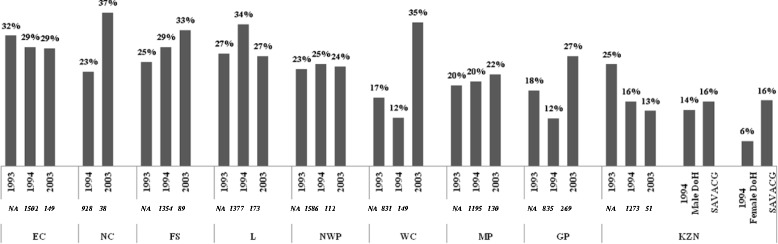
The prevalence of stunting in each of the provinces as determined by three national surveys. EC: Eastern Cape [29, 30, 33]; NC: Northern Cape [29, 32]; FS: Free State [29, 30, 33]; L: Limpopo [29, 30, 33]; NWP: North West Province [29, 30, 33]; WC: Western Cape [29, 30, 33]; MP: Mpumalanga [29, 30, 33]; GP: Gauteng Province [29, 30, 33]; KZN: KwaZulu Natal [29, 30, 33]. Sample size *in italics*, *NA:* Not available. DoH: Department of Health (sample size of male and female = 468 and 440, respectively); SAVACG: The South African Vitamin A Consultative Group (size of male and female = 1037 and 1182, respectively)

Figure 4 shows the prevalence of stunting in each province from the three national surveys that published provincial data in the 1993 Living Standards and Development Survey [30]; the 1994 South African Vitamin A Consultative Group (SAVACG) [29]; and the 2003 Demographic Health Survey (DHS) [32]. Qualitatively, the figure shows that the majority of rural provinces, Eastern Cape (EC), Northwest province and Mpumalanga, have a high but consistent level of stunting in comparison to Gauteng (GP) and Western Cape (WC) that show marked fluctuations. The sample sizes per province were not provided for the 1993 national survey [30]. As a result, it is impossible to assess whether the large differences in prevalence observed between 1993 and 1994, in particular in GP (from 18 % to 12 %), in KwaZulu-Natal (KZN, from 25 % to 16 %), in WC (from 17 % to 12 %) and Limpopo (from 27 % to 34 %), are statistically significant. Between 1994 [29] and 2003 [32], there were significant increases in the prevalence of stunting from 23 % to 37 % in Northern Cape (NC,) (p < 0.05), from 12 % to 35 % in WC (p < 0.001) and from 12 % to 27 % in GP (p < 0.001). In the DHS [32], small sample sizes at provincial level may impact on the representativeness of the estimates and suggests a cautious interpretation. In 1994, the prevalence of stunting found by the SAVACG [29] in KZN were significantly higher than those reported in the Department of Health Primary Schools Survey in the same year [28] (z-test, p < 0.001 and p < 0.001, for boys and girls respectively). The discrepancy could be due to age range differences between the two surveys (SAVACG [29] on 0–5 year-old children, primary schools survey [28] on 4–5 year-old children) as stunting is most prevalent in the first two years of life. In addition, samples may differ between these two studies since the primary school survey did not include primary school age children that did not attend school. In 2003, the provinces with the highest prevalence of stunting were NC, Free State (FS), and WC [32].

Only two of the three national surveys [32, 33], that published ethnicity data, reported the prevalence of stunting in Indians, which is likely to have large confidence intervals considering the limited sample sizes (Additional file 4). The sample sizes were not provided for the 1993 survey [30], therefore it is difficult to assess whether there is statistical differences in the prevalence of stunting in the different ethnic groups between 1993 and 2003. Data from the NIDS [33], reported a higher prevalence of stunting in black and mixed ancestry children than white and Indian children (z-test, p < 0.001 for all comparisons).

At a national level, the prevalence of stunting increased by 10 % between 1994 [29] and 2003 [32] (z-test, p < 0.001) in urban black children, and by 3 % between 1986 [27] and 2003 [32] in rural black children (z-test, p < 0.01) (Additional files 5 and 6). In 2003 [32], there was no statistical difference in the national prevalence of stunting between urban (26.9 %) and rural areas (28 %). The figures show that the prevalence of stunting is not documented for all provinces or for all ethnic groups (black Africans are represented in 80 % of the studies) over time, and that there are disparities between localities within provinces. For studies that used the NCHS standard, the prevalence of stunting reported in the ad hoc studies in rural or urban areas, are in line or slightly higher for particular groups than in the national surveys done around the same year/period (Additional files 5 and 6). However, there were wide ranges reported which suggests that there are pockets or areas where stunting is a greater burden than other areas.

## Discussion

### Temporal changes in the number of studies on stunting in children

In South Africa, between 1960 and 1990, there was a reduction in the importance of undernutrition as a government public health concern [74]. Indeed, the Department of Nutrition (under the Ministry of Health) was disbanded and the activities of the National Nutrition Council, whose mandate was to conduct research in nutrition, slowly declined [74]. However, the increase of publications on stunting during that 30 years period shows that in the meantime stunting was a growing health concern among the academics. At the international level, until 1970, underweight was the main indicator of malnutrition which contributes to explain that there were fewer studies conducted on stunting in children. It is in 1971 that the Joint FAO/WHO meeting [75] emphasized the importance of assessing height in relation to age [24, 76, 77]. In addition, from the 1970s, nutritional epidemiology experienced major changes that improved the evaluation of nutritional status, including the standardization of methods, the homogenization of the indicators and the definition of their cut-off points [24, 25, 78], the creation of the first international growth curves (NCHS) [22], and the use of advanced technology and tools for data analyses. In 1995, the creation of a Directorate of Nutrition (under the Department of Health) and the launch of the Integrated Nutrition Program in 1996 was a sign of the recognition of malnutrition as a national public health concern in South Africa.

In this systematic review we found that the number of publications on stunting reached a peak in the 1990s. Although, the number of publications diminished in the 2000s, six national surveys [16, 30–34] were published providing a regular assessment of the prevalence of stunting. In addition, regional studies have provided supplementary information on malnutrition and the associated ecological factors in high risk areas [62, 65, 67–70].

### Temporal trends in the prevalence of stunting

Our review highlights methodological challenges when trying to understand temporal trends in stunting prevalence. Firstly, differences between growth standards/references only allow for comparisons between prevalence estimates using the same standard/reference, as the prevalence changes according to the growth standard/references used. As reported in other countries [79], the use of the WHO standard has been shown to increase the prevalence of stunting in the South African population [31, 34, 73]. In addition, the cut-off points used to define stunting have varied within these references/standards, which increase the degree of variability of the prevalence estimates. Secondly, we observed fluctuations in the prevalence of stunting across time that may be a result of different sampling methodologies. The increase in the national prevalence of stunting by 6.5 % over a 3-year period (between 2005 and 2008) may be real but differences in sampling methods, that do not capture comparable socio-demographic variability, may also be responsible for some of the difference. Sampling concerns are exacerbated in longitudinal/cross-sectional studies that often target vulnerable populations and as a consequence are not representative of the provinces where the studies were undertaken.

This review also underlines the persistence of stunting and its heterogeneous distribution across the age ranges. Our analysis suggests that the national prevalence compared to the 1993 figure [30] had increased by 3 to 5 % by 2008 [33], but had decreased by 10.5 % by 2013 [30]. Both the NIDS in 2008 and the SANHANES in 2013 used rigorous sampling methodologies [16, 80]. Therefore the large decrease in stunting between these surveys may suggest: (i) trickling down of economic growth that has improved maternal and child nutrition; and (ii) successful reduction in mother-child transmission of HIV. However, the most recent stunting prevalence data of 26.9 % in boys and 25.9 % in girls between 0 and 3 years of age, confirms that stunting persists as a public health burden [16]. Impaired growth during early life has been associated with delays in cognitive development, and an increased risk of obesity and cardio-metabolic disease later in life [11, 81]. The short and long-term adverse effects highlight the importance of tackling stunting early in life to improve human capital globally [11]. The first thousand days of life, from conception to 2 years of age, have been identified as a window of opportunity for interventions to improve childhood growth and development, and reduce future disease risks [82, 83].

### Regional changes in the prevalence of stunting

Our analysis shows that the burden of stunting is not homogeneous across provinces. Between 1994 [30] and 2003 [16], large increases in the prevalence of stunting were noted in the NC, WC and GP. In addition to sampling differences over time, the migration pattern during that period could partly explain that observation. In the 1990’s, labour populations, predominantly from the homelands, migrated to the provincial metropolises of GP and WC where they established informal settlements [84, 85]. Little is known about the pattern of preschool children’s mobility but it could be motivated either by maternal migration or the families searching for better access to child care [86, 87]. The transition from rural homes to the generally poor living conditions in urban shacks may contribute to poor health outcomes in children [86].

Over the last two decades prevalence rates of stunting ≥20 % were reported in the EC, NC, FS, North West province, and Limpopo. These mainly rural provinces have had fewer studies, probably reflecting the lack of resources such as funding opportunities, medical/public health academies and facilities [88]. In addition, these provinces have higher percentages of unemployment and poverty, and informal settlements [89] with poor access to water and sanitation. In South Africa, the National Department of Health is responsible for national health policy and each province is in charge of developing its own implementation of the national policy [90]. Given the provincial differences, cooperation between the provincial health administrations should be encouraged to highlight policies and programs that work.

Stunting is highly correlated with poorer socio-economic status and environmental conditions [30] and despite the efforts to enhance equity since the introduction of democracy in 1994, South Africa still has one of the highest income inequalities with black and mixed ancestry groups being the most impoverished [90]. Surveys show a higher prevalence of stunting in black and mixed ancestry South African children compared to their white and Indian peers. Unlike the findings from other Sub-Saharan African countries [2, 91], no rural–urban [32] or sex [16, 32] differences were found in the prevalence of stunting in under-6 year old children. In fact, urbanization leads to an increase number of inhabitants in informal settlements and, as a consequence, to a rise of poor health outcomes in these areas [16, 92]. Interestingly, when looking at a wider age range (0–14 years), recent surveys [16, 33] report that the highest prevalence of stunting is found in children living in informal settlements of rural and urban areas. Accordingly, differences could exist in the prevalence of stunting in children under-6 years of age within both rural and urban settings in communities with different socio-economic status which is in line with the disparities within provinces which have been observed over time. However, it could not be assessed in this review as these prevalence rates are not provided by recent surveys.

## Conclusions

South Africa is far from reaching the MDG1 [93, 94]. This systematic review indicates that despite economic transition over the past 40 years, stunting still persists at a significant level. This observation suggests that the implementation of the national strategy (the Integrated Nutrition Programme) in 1996 has not reduced the prevalence of early life malnutrition. In that regards, the 2009 Landscape Analysis [95] helped to identify critical restraints and to make nutrition-related recommendations to scale-up the national nutrition programs. For instance, the Directorate of Nutrition (National Department of Health) defined a *Roadmap for Nutrition in South Africa for the period of 2013 – 2017* [96] which advocates for a *multi-sectoral approach* to tackle malnutrition including *a lifecycle approach* focusing on the key ‘window of opportunity’, from pregnancy to two years of age (the first 1000 days). Other low and middle income countries in rapid transition, such as Brazil and India, have adopted a multi-sectoral approach. This approach is characterized by the implementation of interventions against malnutrition that are equity driven, nutrition sensitive and specific to the targeted populations. This multi-sectoral approach has resulted in a significant decrease in the prevalence of stunting. The success of the approach is also due to anchoring interventions into programs of different institutional sectors that are directly or indirectly linked to the issue of malnutrition (e.g., agricultural policies). In Brazil stunting prevalence of children under 5 years of age has decreased from 37.4 % in 1974 to 7.1 % in 2007, and has been attributed to strong political commitments and to improvements in (1) the purchasing power of low-income families; (2) female education levels; (3) public water and sanitation systems; (4) access to basic health care, and (5) the quantity and quality of subsistence crops [9, 97]. In India, the Rajmata Jijau Mother-Child Health and Nutrition Mission [9], which was aimed at promoting behavioral changes, advocating to policy makers for the importance of the first 1000 days; promoting the active participation of the community; and, measuring the impact of the programmes succeeded in halving the prevalence of stunting in children under 2 years of age between 2005 and 2012.

This systematic review recognizes the difficulty in comparing surveys over time, and recommends that South African public health practitioners and policy-makers to: (1) adopt one growth standard for the assessment of stunting; (2) agree on the sampling methodology for national surveys; (3) generate representative data at provincial levels to improve the national sampling framework; and (4) lodge and archive national survey datasets with the Department of Health (or the South African Medical Research Council) such that these data can be reanalyzed if necessary in the future. Addressing these issues would facilitate better monitoring of malnutrition, identify vulnerable communities and individuals, and improve national policies and local programs [8].

The experience of South Africa, the most developed economy in sub-Saharan Africa, highlights the difficulties in tackling malnutrition on the sub-continent. The persistence of early life malnutrition is fueling the burden of non-communicable disease, an emerging public health concern in Sub-Saharan Africa.

## Additional files

Additional file 1:
**PRISMA 2009 Checklist.**
Additional file 2:
**List of the references that could not be procured.**
Additional file 3:
**List of the references included in the systematic review.**
Additional file 4:
**Evolution of national prevalence of stunting by ethnic groups.** Histogram of ethnic prevalence of stunting in children less than 6 years of age. Additional file 5:
**Prevalence of stunting in urban areas per provinces.** Histogram of regional prevalence of stunting in children less than 6 years of age. Additional file 6:
**Changing prevalence of stunting in rural areas: national and black population prevalence.** Histogram of regional prevalence of stunting in children less than 6 years of age.

## Abbreviations

CDC: Centers for Disease Control and prevention
DHS: Demographic Health Survey
EC: Eastern Cape
FS: Free State
GP: Gauteng Province
KZN: KwaZulu Natal
MDG: Millennium Development Goal
NC: Northern Cape
NCHS: National Center for Health Statistics
NHANES I and II: The National Health and Nutrition Examination Survey I and II
NIDS: The National Income Dynamic Study
PRISMA: Preferred Reporting Items for Systematic Reviews and Meta-Analyses
SANHANES-1: The first South African National Health And Nutrition Examination Survey
SAVACG: The South African Vitamin A Consultative Group
SSA: Sub-Saharan Africa
TQS: Total Quality Score
WHO: World Health Organization
WC: Western Cape
