# Supplementary material for: Has the prevalence of stunting in South African children changed in 40 years? A systematic review
Source: BMC Public Health. 2015 Jun 5;15:534. doi: 10.1186/s12889-015-1844-9 (PMC4456716; doi:10.1186/s12889-015-1844-9)
Supplement: Additional file 2: — List of the references that could not be procured. [file 12889_2015_1844_MOESM2_ESM.docx]

**Additional file 2. References not procured**

1. Richardson BD. S Afr J Sci. 1978;74:246.
2. Coovadia HM, Adhikari M, Mthethwa D. Physical growth of Negro children in the Durban area. Trop Geogr Med. 1978;30(3):373-381.
3. Howard VM. Research between 1950 and 1980 on urban-rural differences in body size and growth rate of children and youths. Adv Child Dev Behav. 1982;17:83-138.

1. Kotze JP, van der Merwe GJ, Mosert WP et al. Anthropometric survey of different cultural groups in South Africa. J Dietet Home Econ. 1982;10(2):77-81.
2. Schlemmer L, Stopforth P. A study of malnutrition in the Nqutu district of KwaZulu. KwZulu Natal: Institute for Social Research, University of Natal; 1974. Fact Paper No. 2.
3. Department of Health and Welfare. The Inanda Nutrition Survey. Pretoria, South Africa: Department of Health and Welfare; 1984. Epidemiological comments Volume 11 Number 4 and 7.
4. Fincham RJ. A household survey into the nutritional status of rural black pre-school children in the dias divisional area of the Eastern Cape. Eastern Cape, South Africa: Institute of Social and Economic Research, Rhodes University; 1982. Working Paper No 6.
5. Fincham RJ. The nutritional status of pre-school children in the Amatola Basin, Eastern Cape. Eastern Cape, South Africa: Institute of Social and Economic Research, Rhodes University; 1982. Working Paper No 9.
6. Fincham RJ. Nutritional status of black communities in the Eastern Cape: South Africa - assessment and policy recommendations. In: Rathwell T, Phillips D, eds. Health, Race and Ethnicity. Beckenham, Kent: Croom Helm; 1986.
7. Burgess JD, Ireland JD, Hoogenhout DH. Practical considerations in malnutrition. Nurs RSA. 1988;3(7):28-31.
8. Wagstaff L, Reinach SG, Richardson BD, Mkhasibe C, de Vries G. Anthropometrically determined nutritional status and the school performance of black urban school children. Hum Nutr Clin Nutr. 1987;41:277-286.
9. Walker ARP, Walker BF, Jones J, Kadwa M. Growth of South African Indian schoolchildren in different social classes. J R Soc Health. 1989;2:54-56.
10. Yach D, Coetzee N, Hugo-Hamman CT, Fisher SA, Kibel MA. Identifying children at risk in peri-urban Cape Town. S Afr J Epidemiol Infect. 1990;5:6-8.
11. Setswe G. Prevalence and risk factors for malnutrition among children aged 5 years and less in the Lefaragatlha village of Bophuthatswana. Curationis. 1994;17(3):33-35.
12. Walker AR, Walker BF, Glatthaar II. Is the lesser growth of African schoolchildren essentially prejudicial to their present and future health? Trop Geogr Med. 1995;47(4):145-150.
13. Den Besten L, Bac M, Glatthaar II, Walker ARP. Changes in the anthropometric status of rural African under-fives during a decade of primary health care. J Trop Med Hyg. 1995;98:361-366.
14. Steyn K, Bourne L, Jooste P, Fourie JM, Rossouw K, Lombard C. Anthropometric profile of a black population of the Cape Peninsula in South Africa. East Afr Med J. 1998;75(1):35-40.
15. Hendriks M, Fernandes M, le Roux M, Hussey G. An evaluation of the protein energy malnutrition scheme in the Northern Cape Province of South Africa. S Afr Med J. 1998;88(6)(Suppl):3.
16. Faber M, Oelofse A, Benade AS. A model for community-based growth monitoring system. Afr J Health Sci. 1998;5(1-2):72-78.
17. Oelofse A, Van Raaij JM, Benade AJ, Dhansay MA, Tolboom JJ, Hautvast JG. The effect of a micronutrient-fortified complementary food on micronutrient status, growth and development of 6- to 12-month-old disadvantaged urban South African infants. Int J Food Sci Nutr. 2003; 54(5):399-407.
18. Faber M, Swanevelder S, Benadé AJS. Is there an association between the nutritional status of the mother and that of her 2-year-old to 5-year-old child? Int J Food Sci Nutr. 2005; 56(4):237-244.
