# Supplementary material for: Has the prevalence of stunting in South African children changed in 40 years? A systematic review
Source: BMC Public Health. 2015 Jun 5;15:534. doi: 10.1186/s12889-015-1844-9 (PMC4456716; doi:10.1186/s12889-015-1844-9)
Supplement: Additional file 3: — List of the references included in the systematic review. [file 12889_2015_1844_MOESM3_ESM.docx]

**Additional File 3. Table of References**

| **Authors [Reference number]** | **Year (publication or survey)** | **Provinces** | **Location** | **Specificity of the area** | **Rural-Urban** | **Setting** | **Ethnicity** | **Age range (years)** | **Growth Curves** | **Cut-off point** | **Total quality score** |
| --- | --- | --- | --- | --- | --- | --- | --- | --- | --- | --- | --- |
| **Shuenyane et al., 1977 [35]** | 1975 | Gauteng Province | Diepkloof - SOWETO Johannesburg | Township | Urban | Cross-sectional | Black | 2-5 | Harvard | <3rd percentile | 9 |
| **Margo et al., 1976 (1) [14]** | 1976 | Gauteng Province | Muldersdrift (30 km N Johannesburg) | Semi-rural | Rural | Cross-sectional | Black | 1-6 | Harvard | <3rd percentile | 10 |
| **Margo et al., 1976 (2) [15]** | 1976 | Gauteng Province | Western Township (Johannesburg) | Township | Urban | Cross-sectional | Coloured | 1-4 | Harvard | <3rd percentile | 10 |
| **Westcott and Stott, 1977 [36]** | 1977 | Eastern Cape | Jecweni - Transkei | Homeland | Rural | Cross-sectional | Black | 1.5-3 | Harvard | <3rd percentile | 7 |
| **Richardson, 1980 [37]** | 1980 | Gauteng Province | Johannesburg | Township and Johannesburg | Urban | Cross-sectional | Black - Coloured - White | 0-6 | Harvard | ≤90% median | 9 |
| **Adhikari and Coovadia, 1981 [38]** | 1981 | KwaZulu Natal | Durban |  | Urban | Cross-sectional | Black | 1-5 | Harvard | <3rd percentile | 6 |
| **Krynauw et al., 1983 [39]** | 1981 | Eastern Cape | Port Elizabeth |  | Rural | Cross-sectional | Black | 0-5 | NCHS | <3rd percentile | 15 |
| **Lazarus and Bhana, 1984 [41]** | 1981 | KwaZulu Natal | Tongaat, South Area (22 km Durban) |  | Urban | Cross-sectional | Indian | 2-5 | NCHS | Between 5th-10th percentile, <5th percentile | 11 |
| **Ndlovu, 1984 [40]** | 1982 | KwaZulu Natal | Esikhani towonship and Mpukonyoni | Township and Rural | Urban and Rural | Cross-sectional | Black | 0-5 | Harvard | <80% median | 5 |

| **Authors [Reference number]** | Year | Provinces | Location | Specificity of the area | Rural Urban | Setting | Ethnicity | Age range (years) | Growth Curves | Cut-off point | Total quality score |
| --- | --- | --- | --- | --- | --- | --- | --- | --- | --- | --- | --- |
| **Richardson, 1986 [43]** | 1981/1983 and 1985 | Rural Transvaal and Gauteng Province | Rural Transvaal and Johannesburg |  | Rural and Urban | Cross-sectional | Black-Coloured-White-Indian | 1-5 |  | ≤90% median | 3 |
| **Richardson and Sinwel, 1984 [42]** | 1984 | North West Province | Gelukspan Bophuthatswana | Homeland | Rural | Cross-sectional | Black | 0-0.6 | NCHS | < 90% median | 8 |
| **Grant, 1994 [57]** | 1984 | Gauteng Province and North West Province |  | N/A | Urban and Rural |  | White - Indian - Black | 4-5 |  | <5th percentile | N/A |
| **Househam and Elliott, 1987 [44]** | 1985 | Free State | Bloemfontein |  | Urban | Cross-sectional | Black | 0.5-4 | NCHS | ≤5th percentile | 4 |
| **Hugo-Hamman et al., 1987 [45]** | 1986 | Western Cape | Khayelitsha (Site C) | Township | Urban | Cross-sectional | Black | 1-5 | NCHS | ≤3rd percentile | 19 |
| **RHOSA, 1987 [27]** | 1986 | National | Free State -Gauteng Province - Limpopo - KwaZulu Natal - Western Eastern Northern Cape |  | Rural | Cross-sectional | Black | 0-4.9 | NCHS | <3rd percentile | 15 |
| **Jacobs et al., 1988 [46]** | 1987 | Western Cape | Mamre | Farming community | Rural | Cross-sectional | Coloured | 3-6 | NCHS | <3rd percentile | 12 |
| **Barron et al., 1991 [50]** | 1987 | Gauteng Province | Oukasie | Township | Urban | Cross-sectional | Black | 0-5 | NCHS | <-2 SD | 12 |

| **Authors [Reference number]** | **Year** | **Provinces** | **Location** | **Specificity of the area** | **Rural Urban** | **Setting** | **Ethnicity** | **Age range (years)** | **Growth Curves** | **Cut-off point** | **Total quality score** |
| --- | --- | --- | --- | --- | --- | --- | --- | --- | --- | --- | --- |
| **Le Roux and Le Roux, 1991 [48]** | 1988 | Western Cape | Khayelitsha (Site B) | Township | Urban | Cross-sectional | Black | 0-6 | NCHS | <90% of expected height for age | 7 |
| **Byarugaba, 1991 [47]** | 1990 | Eastern Cape | Transkei | Homeland | Urban - Periurban - Rural | Cross-sectional | Black | 0-5 | NCHS | ≤3rd percentile | 16 |
| **Ramphele, Heap and Trollip, 1991 [49]** | 1990 | Western Cape | Langa (The Zones) | Township | Urban | Cross-sectional | Black | 0-6 | NCHS | ≤3rd percentile | 15 |
| **Bourne et al., 1994 [54]** | 1990 | Cape Town |  | N/A | Urban | Cross-sectional | Black | 3-6 | NCHS | ≤-2SD | N/A |
| **Coetzee and Ferinho, 1994 [52]** | 1990 | Gauteng Province | Alexandra | Township | Urban | Cross-sectional | Black | 1-2 | NCHS | ≤3rd percentile | 17 |
| **Steyn et al., 1992 [51]** | 1990/1991 | Limpopo |  | Homeland | Rural |  | Black | 3-5 |  | <5th percentile | N/A |
| **Coutsoudis et al., 1994 [56]** | 1991 | KwaZulu Natal | Besters | Township | Urban | Cross-sectional | Black | 3-6 | NCHS | <3rd percentile | 12 |
| **Solarsh et al., 1994 [53]** | 1992 | KwaZulu Natal | Nqutu district | N/A | Rural | Cross-sectional | Black | 0.5-6 | NCHS | <-2SD | 11 |
| **Walsh, 1995 [59]** | 1992 | Free State and Northern Cape |  | N/A | Rural and Urban |  | Coloured | 2-6 |  | Between -3SD and  -2SD | N/A |
| **Steyn et al., 1994 [55]** | 1993 | Limpopo | Monyamane (45 km Polokwane) | N/A | Rural | Cross-sectional | Black | Preschools | NCHS | <-2SD | N/A |
| **Wagstaff et al., 1994 [58]** | 1994 | Gauteng Province | Greater Johannesburg-Soweto | N/A | Urban | Cross-sectional | Black | 2.5-4 |  | <80% median | 4 |
| **DoH, 1994 [28]** | 1994 | KwaZulu Natal | N/A | N/A | Overall | Cross-sectional | Overall | 4-5 | NCHS | <-2SD | 6 |

| **Authors [Reference number]** | **Year** | **Provinces** | **Location** | **Specificity of the area** | **Rural Urban** | **Setting** | **Ethnicity** | **Age range (years)** | **Growth Curves** | **Cut-off point** | **Total quality score** |
| --- | --- | --- | --- | --- | --- | --- | --- | --- | --- | --- | --- |
| **Zere and Mc Intyre, 2003 [30]** | 1993 | National | Eastern Cape - Free State - Gauteng Province - KwaZulu Natal - Mpumalanga - North West province - Limpopo - Western Cape | N/A | Overall - Rural -Urban - Metropolitan | Cross-sectional | Overall - Black - Coloured - White | 0-5 | NCHS | <-2SD | 18 |
| **SAVACG 1995 [29]** | 1994 | National | Northern Cape - Western Cape - Eastern Cape - Limpopo -Gauteng Province - North West Province - Free State – Mpumalanga- KwaZulu Natal | N/A | Rural-Urban | Cross-sectional | Overall | 0.5-6 | NCHS | <-2SD | 20 |
| **Norris et al., 2009 [73]** | 1990-1995 | Gauteng Province | SOWETO | Township | Urban | Cross-sectional / Longitudinal | Overall | 0.5-5 | NCHS - CDC - WHO | <-2SD | 15 |
| **Dannhauser et al., 1996 [60]** | 1996 | Free State |  | N/A | Rural | [Not precised] | Black | 0.5-6 |  | <-2SD | N/A |
| **Monyeki et al., 2000 [63]** | 1996 | Limpopo | Ellisras | N/A | Rural | Cross-sectional | Black | 3-5.9 | NHANES I and II | <-2SD | 11 |
| **Oelofse et al., 2002 [66]** | 1998 | Western Cape | Cloetesville and Kayamandi | N/A | Urban | Cross-sectional | Black and Coloured | 0.5-1 | NCHS | - 2SD | 5 |
| **Oelofse et al., 1999 [61]** | 1999 | KwaZulu Natal | Ndunakazi | N/A | Rural | Cross-sectional | Black | 0.5-5 | NCHS | <-2SD | 8 |
| **Steyn et al., 2005 [31]** | 1999 | National | Eastern Cape - Free State - Gauteng Province - KwaZulu Natal - Mpumalanga - North West province - Limpopo - Western Cape - Northern Cape | N/A | Overall | Cross-sectional | Overall | 1-6 | NCHS | ≤-2SD | 20 |

| **Authors [Reference number]** | **Year** | **Provinces** | **Location** | **Specificity of the area** | **Rural Urban** | **Setting** | **Ethnicity** | **Age range (years)** | **Growth Curves** | **Cut-off point** | **Total quality score** |
| --- | --- | --- | --- | --- | --- | --- | --- | --- | --- | --- | --- |
| **Dannahauser et al., 2000 [62]** | 2000 | Free State | Joe Slovo and JB Mafora (Mangaung) | Township | Urban | Cross-sectional | [Not precised] | 0-6 | NCHS | <-2SD | 8 |
| **Cleaton-Jones et al, 2000 [64]** | 2000 | North West Province and Gauteng Province | Gelukspan (Bophuthatswana), Soweto, Lenasia, Johannesburg | Township and town | Rural and Urban | Cross-sectional | White-Black-Indian | 4-5 | NCHS | Between 95 - <85% of the median | 8 |
| **Walsh et al., 2002 [65]** | 2002 | Free State and Northern Cape | Heidedal and Ritchie - Jagersfontein, Fauresmith - Trompsburg - Bethulie | Townships | Urban and Rural | Cross-sectional | [Not precised] | 2-5 | NCHS | < -2SD | 7 |
| **Mamabolo et al., 2005 [72]** | 2002-2003 | Limpopo | [Not precised] | N/A | Rural | Longitudinal | Black | 3 | NCHS | <-2SD | 13 |
| **Chopra, 2003 [67]** | 2003 | KwaZulu Natal | Hlabisa | N/A | Rural | Cross-sectional | Black | 0.25-5 | NCHS | <-2SD | 16 |
| **Smuts et al. 2008 [70]** | 2003 | Eastern Cape and KwaZulu Natal | OR Tambo - Alfred Nzo districts and Umkhayakude - Zululand district | N/A | Rural | Cross-sectional | Black | 0-5 | NCHS | <-2SD | 13 |
| **DHS 2003 [32]** | 2003-2004 | National | Eastern Cape - Northern Cape - Free State - Gauteng Province - KwaZulu Natal - Mpumalanga - North West province - Limpopo - Western Cape | N/A | Overall - Rural - Urban | Cross-sectional | Black - Coloured - White - Indians | 0-5 | NCHS | <-2SD | 17 |
| **Kleynhans et al., 2006 [68]** | 2006 | Limpopo and  Gauteng Province | Sekuruwe - Molekane and Atteridgeville | N/A | Rural and Urban | Cross-sectional | Black | 1-2 | NCHS | <-2SD | 13 |

| **Authors [Reference number]** | **Year** | **Provinces** | **Location** | **Specificity of the area** | **Rural Urban** | **Setting** | **Ethnicity** | **Age range (years)** | **Growth Curves** | **Cut-off point** | **Total quality score** |
| --- | --- | --- | --- | --- | --- | --- | --- | --- | --- | --- | --- |
| **Faber and Benade, 2007 [69]** | 2007 | KwaZulu Natal | Valley of a Thousand Hills | N/A | Rural | Cross-sectional | Black | 0.6-1 | NCHS | <-2SD | 11 |
| **Kruger et al., 2012 [34]** | 1999 and 2005 | National | Eastern Cape - Northern Cape - Free State - Gauteng Province - KwaZulu Natal - Mpumalanga - North West province - Limpopo - Western Cape | N/A | Overall-Rural-Urban | Cross-sectional | Overall | 1-6 | WHO | <-2SD | 19 |
| **Kimani-Murage et al., 2010 [71]** | 2007 | Mpumalanga | Agincourt sub-district | N/A | Rural | Cross-sectional | Black | 1-4 | WHO | <-2SD | 16 |
| **Ardington and Case, 2009 [33]** | 2008 | National | Eastern Cape - Northern Cape - Free State - Gauteng Province - KwaZulu Natal - Mpumalanga - North West province - Limpopo - Western Cape | N/A | Overall | Cross-sectional | Overall | 0.5-5 | WHO | <-2SD | 15 |
| **SANHANES, 2014 [16]** | 2013 | National | Eastern Cape - Northern Cape - Free State - Gauteng Province - KwaZulu Natal - Mpumalanga - North West province - Limpopo - Western Cape | N/A | Overall | Cross-sectional | Overall | 0-6 | WHO | <-2SD | 20 |
