# Supplementary material for: Has the prevalence of stunting in South African children changed in 40 years? A systematic review
Source: BMC Public Health. 2015 Jun 5;15:534. doi: 10.1186/s12889-015-1844-9 (PMC4456716; doi:10.1186/s12889-015-1844-9)
Supplement: Additional file 4: — Evolution of national prevalence of stunting by ethnic groups. Histogram of ethnic prevalence of stunting in children less than 6 years of age. [file 12889_2015_1844_MOESM4_ESM.docx]

**Additional file 4. Evolution of national prevalence of stunting by ethnic groups**

**2008**

***3201 284 226 87***

**Black MA White Indian**

**WHO**

References: Integrated Household Survey^30^ (1993); Demographic and Health Survey^32^ (2003); National Income Dynamic Survey^33^ (2008)

Sample size *in italics*, *NA*: Not available, MA: mixed ancestry.
