# Supplementary material for: Has the prevalence of stunting in South African children changed in 40 years? A systematic review
Source: BMC Public Health. 2015 Jun 5;15:534. doi: 10.1186/s12889-015-1844-9 (PMC4456716; doi:10.1186/s12889-015-1844-9)
Supplement: Additional file 5: — Prevalence of stunting in urban areas per provinces. Histogram of regional prevalence of stunting in children less than 6 years of age. [file 12889_2015_1844_MOESM5_ESM.docx]

**Additional file 5. Prevalence of stunting in urban areas per provinces**

EC: Eastern Cape; FS: Free State; WC: Western Cape; GP: Gauteng Province; KZN: KwaZulu Natal. MA: Mixed Ancestry.

National Black 2003

1990

Black

MA

Black

MA

White

1980

Black

Indian

White

2000

References per growth reference/standards (left to right)

Boston growth reference: 35, 15, 37 – 38, 40

NCHS growth reference: 30, 29, 32 – 41, 56 – 45, 48, 54, 49, 66 – 50, 52, 73, 64, 68 – 44, 62, 65 - 47

CDC and WHO growth standards: 73
