# Supplementary material for: Has the prevalence of stunting in South African children changed in 40 years? A systematic review
Source: BMC Public Health. 2015 Jun 5;15:534. doi: 10.1186/s12889-015-1844-9 (PMC4456716; doi:10.1186/s12889-015-1844-9)
Supplement: Additional file 6: — Changing prevalence of stunting in rural areas: national and black population prevalence. Histogram of regional prevalence of stunting in children less than 6 years of age. [file 12889_2015_1844_MOESM6_ESM.docx]

**Additional file 6. Changing prevalence of stunting in rural areas: national (overall population) and black population prevalence**

References per growth curve (left to right)

**L**

**1996**

**MP**

**2007**

**GP FS**

**1986**

**NATIONAL**

**1986 1993 1994 2003**

**National Black**

**2003**

**L**

**1986 1993 2005**

**NWP**

**1984 2000**

**KZN**

**1986 1999 2003 2007**

Boston growth reference: 36, 40, 14

NCHS growth reference: 26, 31, 29, 32 – 26, 61, 70, 67, 69 – 39, 26, 47, 70 – 42, 64 – 30, 55, 68, 72

NHANES growth reference: 63

WHO growth standard: 71

Provinces

FS: Free State; L: Limpopo; NWP: North West Province; MP: Mpumalanga; GP: Gauteng Province; KZN: KwaZulu Natal

NATIONAL

Overall South African population for the surveys in 1993, 1994, 2003; overall black South African in the survey in 1986
